# The epithelial transcriptome and mucosal microbiota are altered for goats fed with a low-protein diet

**DOI:** 10.3389/fmicb.2023.1237955

**Published:** 2023-09-04

**Authors:** Jian Wu, Changxin Tian, Jinzhen Jiao, Qiongxian Yan, Chuanshe Zhou, Zhiliang Tan

**Affiliations:** ^1^CAS Key Laboratory of Agro-ecological Processes in Subtropical Region, Institute of Subtropical Agriculture, Chinese Academy of Sciences, Changsha, Hunan, China; ^2^National Engineering Laboratory for Pollution Control and Waste Utilization in Livestock and Poultry Production, Institute of Subtropical Agriculture, Chinese Academy of Sciences, Changsha, Hunan, China; ^3^Hunan Provincial Key Laboratory of Animal Nutritional Physiology and Metabolic Process, Institute of Subtropical Agriculture, Chinese Academy of Sciences, Changsha, Hunan, China; ^4^University of Chinese Academy of Sciences, Beijing, China

**Keywords:** immune homeostasis, low protein diet, microbiome, transcriptome, goat

## Abstract

**Introduction:**

Feeding low protein (LP) diet to animals impose severe challenge to animals' immune homeostasis. However, limited knowledge about the underlying adaption mechanism of host and ruminal microbiota responding to LP diet were well understood. Herein, this study was performed to examine the changes in relative abundance of ruminal microbiota and host ruminal mucosal transcriptome profiles in response to a LP diet.

**Methods:**

A total of twenty-four female Xiangdong balck goats with similar weight (20.64 ± 2.40 kg) and age (8 ± 0.3 months) were randomly assigned into two groups, LP (5.52% crude protein containing diet) and CON (10.77% crude protein containing diet) groups. Upon completion of the trial, all goats were slaughtered after a 16-hour fasting period in LiuYang city (N 28°15′, E 113°63′) in China. HE staining, free amino acids measurement, transcriptome analysis and microbiome analysis were applied to detect the morphology alterations, free amino acids profile alterations and the shift in host ruminal mucosal transcriptome and ruminal microbiota communities.

**Results:**

Firstly, the results showed that feeding LP diet to goats decreased the rumen papilla width (*P* = 0.043), surface area (*P* = 0.013) and total ruminal free amino acids concentration (*P* = 0.016). Secondly, microbiome analysis indicated that 9 microbial genera, including *Eubacterium* and *Prevotella*, were enriched in LP group while 11 microbial genera, including *Butyrivibrio* and *Ruminococcus*, were enriched in CON group. Finally, in terms of immune-related genes, the expression levels of genes involved in tight junction categories (e.g., MYH11, PPP2R2C, and MYL9) and acquired immunity (e.g., PCP4 and CXCL13) were observed to be upregulated in the LP group when compared to the CON group.

**Conclusion:**

Under the LP diet, the rumen exhibited increased relative abundance of pathogenic microbiota and VFA-degrading microbiota, leading to disruptions in immune homeostasis within the host's ruminal mucosa. These findings indicate that the ruminal microbiota interacts with host results in the disruption in animals' immune homeostasis under LP diet challenge.

## 1. Introduction

Feeding low-protein (LP) diets to goats represent a common strategy for reducing the cost of breeding and nitrogen losses to the environment (Gebeyew et al., 2021). However, as the amino acids play a vital role in maintaining the procedure of animal growth and act as the basal component of the immunity system of the body, low-protein content in the feed imposes negative effects on ruminants' growth performance and homeostasis of the gastrointestinal tract (GIT) (Wang et al., 2021). Furthermore, inadequate protein supply for animals is closely linked to metabolic and immune disorders, such as inflammatory bowel diseases and cancer development (Lan et al., 2015; Soares et al., 2020).

Numerous microorganisms colonize the GIT of mammals and play vital roles in the procedures of extracting energy from diets, regulating the homeostasis of the immune system and producing hormones to regulate a series of body activities (Macpherson et al., 2017; Shanahan et al., 2017). Various factors, including diet composition, host genetics, and antibiotics, have been reported to impose significant effects on the diversity of microbiota in the GIT, and finally may impose severe challenges to host health (Gilbert et al., 2018). In humans and mice, a lower protein diet decreased the abundance of *Bifidobacterium* in gut digesta, which produced antimicrobial peptides to protect the gut from pathogenic bacteria infection and decreased the risk of gut inflammation (Liu et al., 2014; Feng et al., 2019). In addition to the microbiota residing in the digesta, the microbiota colonizing at the mucosal border is also vital to maintain host metabolic and immune homeostasis (Jiao et al., 2018). Despite the significance, its interactions with the host are still not well interpreted.

As the rumen represents the first site for interplay among ingested nutrients, host GIT, and the microbiota, maintaining metabolic, and immune homeostasis in the rumen is of great importance to ruminants. In lambs and beef steers, limited protein supply caused drastic alterations in bacteria diversity and fermentation parameters in the rumen digesta (Wang et al., 2017; Lv et al., 2020). Thus, we hypothesized that the ruminal mucosal microbiota of low-protein diets would be different from that of control diets, and such a variation could manipulate the molecular adaptation of the rumen. To validate this, we used a combination of 16S rRNA sequencing and mucosal transcriptome analysis to dissect bacterial diversity and the host responses in the rumen mucosa.

## 2. Materials and methods

### 2.1. Animals, housing, and experimental design

All procedures for animal experimentation were performed according to the protocol ISA-2019-0115 approved by the Animal Care Committee, Institute of Subtropical Agriculture, Chinese Academy of Sciences, Changsha, Hunan, China.

A total of 24 female Xiangdong black goats with similar weight (20.6 ± 2.4 kg) and age (8.0 ± 0.3 months) were used in this experiment (Gebeyew et al., 2021). These goats were randomly assigned into two groups, a control (CON) group and a low-protein (LP) group. The experimental diets were designed according to the feeding standards of goats in China (NY/T 816-2004) and met 1.3 times the maintenance requirement of metabolic energy (ME) on the basis of previous studies (Tang et al., 2019), and consisted of 70% of rice straw and 30% of concentrate. The detailed ingredients of experimental diets are shown in Supplementary Table 1. The low-protein diet was formulated to contain half of the CP content of the control diet, and this was achieved by replacing soybean meal with corn meal. The total feeding trial period was 70 days, including 25 days of adaptation and 45 days of experiments. These goats were maintained individually in metabolic cages with access to fresh water offered in equal portions at 07:00 and 17:00 h daily.

### 2.2. Sample collection

After the trial, all goats were slaughtered following a 16-h fasting period. Immediately after all the goats were killed by bleeding the jugular vein by a registered veterinarian in Liuyang city (N 28°15′, E 113°63′), China, ruminal tissues were collected from the ventral sac of the rumen and washed by cold PBS for three times. Afterward, the mucosa samples were separated from the underlying muscular layer, cut into 1 × 1 cm fragments, frozen in liquid nitrogen, and stored at −80°C for subsequent DNA extraction, transcriptome analysis, and free amino acid profile measurements. Moreover, two 2 × 2 cm mucosa samples from the ventral sac of the rumen were collected and fixated in formaldehyde for further HE staining analysis.

### 2.3. HE staining

After fixation in formaldehyde for 24 h, ruminal mucosa was embedded in paraffin wax (Sangon, Shanghai, China) and then successively sliced into 10 pieces of 4–5 μm thick sections followed by mounting onto poly-L-lysine-coated glass slides (Hailun, Changsha, China). The slides were stained with hematoxylin for 5 min followed by immersing the slides into hydrochloric acid and ammonia water for 10 s separately. After washing with running water for 1 h, all the slides were stained with eosin for 5 min. All the images were acquired under a 10× objective lens and a 10× ocular lens by a fluorescence microscope (Olympus, Tokyo, Japan) equipped with DP2-BSW software.

### 2.4. Free amino acid profile

The free amino acid profiles of ruminal mucosa were determined using the procedures described in previous studies (Wu et al., 2020). In brief, 0.4 g of ruminal mucosa was weighed and ground into powder with liquid nitrogen. The free amino acids were extracted from the mucosa by adding 2 ml of 8% sulfosalicylic acid to the mucosa powder. After quiescence at 4°C for 12 h, the samples were centrifuged at 8,000 × g at 4°C for 10 min, and the supernatant fluid was collected and filtered through a 0.22-μm membrane. The samples were analyzed by an automatic amino acid analyzer (L-8900; Hitachi Global Inc., Hitachi, Japan).

### 2.5. Transcriptome analysis

Total RNA was extracted from ruminal mucosa following the instructions of the TaKaRa MiniBEST Universal RNA Extraction Kit (TaKaRa, Dalian, China; code no. 9767). The extracted RNA was reverse-transcribed to build the cDNA library using an mRNA-Seq Sample Preparation Kit (Illumina, San Diego, USA). After verifying and quantifying the cDNA, the libraries were sequenced on the Illumina HiSeq 4000 platform.

The procedures for filtering raw sequencing data were detailed in previous studies (Jiao et al., 2019), and the cleaned data were stored in FASTA format. HISAT2 (v2.04) was applied to map the clean reads to the reference genome (Kim et al., 2015). The clean reads were aligned to the reference coding gene set by Bowtie (Langmead and Salzberg, 2012), and the expression level was measured by RSEM (Li and Dewey, 2011). The analysis of differentially expressed genes (DEGs) was carried out using DESeq2, using thresholds with false discovery rate (FDR) <0.05 and an absolute value of fold change >1.2.

### 2.6. Microbiome analysis

The total DNA of the ruminal mucosa was extracted using the bead-beating method, as detailed in our previous study (Jiao et al., 2015). The quantity of total DNA was measured by NanoDrop ND1000 (NanoDrop Technologies, Inc., Wilmington, DE, USA), and the quality of total DNA was observed by gel electrophoresis. Four samples (1/CON, 3/LP) exhibited significant degradation, failing to meet the standards for library sequencing. After amplifying the V3-V4 region of 16S rRNA (341F, ACTCCTACGGGAGGCAGCAG and 806R, GGACTACHVGGGTWTCTAAT) and running an agarose gel, the bands were purified with a QIAquick Gel Extraction Kit (Qiagen, Hilden, Germany), which was prior to sequencing by the Illumina MiSeq PE250 platform.

The QIIME (Quantitative Insights into Microbial Ecology) pipeline was utilized to perform quality control on the raw data, with a default quality threshold of 20 (Zhang et al., 2019). The quality control process involved a sliding window approach to identify low-quality reads alongside a length screening where reads longer than 200 bases were truncated to 200 bases. Afterward, the short reads were assembled into tags using FLASH, and tags were clustered into amplicon sequence variants (ASVs) of 99% similarity using the unoise3 command implemented in USEARCH (Edgar, 2010). The representative sequences were kept with a total sequencing count >3, and the ASVs were retained that were present in at least two sample replicates to mitigate incidental factors. Taxonomic assignments were performed against the RDP database with a 0.80 confidence threshold (Wang Q. et al., 2007). Alpha and beta diversities were analyzed using the QIIME pipeline. The Bray–Curtis similarity index was used to calculate the distance matrix for principal coordinate analysis (PCoA). The significance of grouping in the PCoA plots was tested by analysis of dissimilarity (ADONIS) with 999 permutations. To identify the differential microbial communities in the ruminal mucosa between these two groups, a linear discriminate analysis (LDA) effect size (LEfSe) method (Segata et al., 2011) was applied with an LDA threshold value of 2.0 using the R software (version 4.0.5).

### 2.7. Statistical analysis

Data were carried out with a one-way analysis of variance (ANOVA) using the R software (version 4.0.5). Student's *t*-test was applied to determine the effects of a low-protein diet on the ruminal mucosal morphology and free amino acid profiles, with a *P*-value of < 0.05 considered significant.

## 3. Results

### 3.1. Morphological analysis

As shown in Table 1, feeding LP diet to goats significantly decreased ruminal papilla width (*P* = 0.043) and surface area (*P* = 0.013), while the length of ruminal papilla showed no significant difference (*P* = 0.268) between these two groups.

**Table 1:** Morphological changes of the rumen papilla in goats fed with LP vs. CON diet.

**Items**	**Treatments**		**SEM**	***P*** **-value**
	**CON**	**LP**		
Papilla length (cm)	1.18	1.16	0.38	0.268
Papilla width (cm)	0.48	0.40	0.09	0.043
Papilla area (cm^2^)	0.60	0.47	0.17	0.013

LP, low-protein group; CON, control group.

### 3.2. Free amino acid profile

Feeding LP diet to goats significantly decreased the total amino acid concentration (*P* = 0.016). The concentration of glutamic acid (*P* = 0.011) and aspartic acid (*P* = 0.037) was significantly decreased in the ruminal mucosa under the LP diet. Similarly, feeding LP diet to goats significantly decreased the concentration of isoleucine (*P* = 0.033) and valine (*P* = 0.040). The concentration of leucine tended to decrease in the ruminal mucosa under the LP diet (*P* = 0.061) (Table 2).

**Table 2:** Effects of different dietary protein levels on rumen mucosa and free amino acid profiles in goats.

**Items (μg/g)**	**Treatments**		**SEM**	***p*-value**
	**CON**	**LP**		
Aspartic acid	46.52	36.65	12.12	0.037
Threonine	123.04	109.20	25.36	0.121
Serine	13.37	12.63	4.21	0.356
Glutamic acid	414.11	328.99	83.74	0.011
Glycine	243.55	223.06	39.91	0.136
Alanine	49.74	41.79	9.361	0.030
Cysteine	2.13	2.53	1.43	0.277
Valine	22.06	18.21	5.39	0.040
Methionine	3.93	3.78	1.35	0.408
Isoleucine	8.71	7.04	2.01	0.033
Leucine	14.17	12.07	2.96	0.061
Tyrosine	11.33	10.75	1.85	0.25
Phenylalanine	10.14	8.42	1.85	0.252
Lysine	16.18	13.44	4.21	0.079
Histidine	1.76	9.66	1.55	0.064
Arginine	8.73	6.75	3.74	0.128
Proline	23.09	21.08	5.89	0.230
NEAA	809.98	644.87	150.64	0.005
EAA	211.63	184.81	36.54	0.044
BCAA	42.18	35.44	9.59	0.06
TAA	1021.61	866.07	161.35	0.016

TAA, total amino acids; BCAA, branch chain amino acids; EAA, essential amino acids; NEAA, non-essential amino acids; LP, low-protein group; CON, control group.

### 3.3. Transcriptome profile and functional analysis

The transcriptome results showed that 41 differentially expressed (DE) genes were observed between the LP and CON groups, of which the expression of 28 genes was upregulated, while 13 genes were downregulated in the LP group in comparison with the CON group. The majority of DEGs were related to immune and muscle categories. For immune-related genes, the expression of genes involved in the categories of tight junction such as *MYH11, PPP2R2C*, and *MYL9*, cancer development such as *TAGLN* and *CALD1*, and acquired immunity such as *PCP4* and *CXCL13* was upregulated in the LP group when compared with that in the CON group (Table 3). For muscle-related genes, the expression of genes involved in the categories of muscle contraction such as *LMOD1, SYNM, CNN1, TPM2, MYH11, MYL9, MYLK*, and *TPM1*, muscle cell proliferation such as *PDLIM3 and RBPMS2*, and muscle development such as *LDB3* and *DMPK* was upregulated in the LP group compared with that in the CON group (Table 4). The expression of gene encoding desmin, which acts as a biomarker of muscle, was downregulated in the LP group when compared with that in the CON group.

**Table 3:** Expression profile of DE immune-related genes.

**Gene symbol**	**Gene name**	**Category**	**logFC (LP vs. CON)**	**Ln *(p*-value)**
MYH11	myosin-11 isoform X2	Tight junction cancer development	2.16	−97.6
PPP2R2C	Serine/threonine-protein phosphatase 2A 55 kDa regulatory subunit B gamma isoform	Tight junction	−1.88	−57.5
MYL9	Myosin regulatory light polypeptide 9 isoform X1	Tight junction	2.11	−91.6
MYLK	Myosin light chain kinase, smooth muscle	Organ damage	2.26	−106.27
PCP4	Purkinje cell protein	Anti-virus	1.46	−28.2
CALD1	Caldesmon isoform X1	Inflammation Stomach cancer	1.36	−40.3
CXCL13	C-X-C motif chemokine 13	Acquired immunity (T cell, B cell)	1.46	−38.3
TAGLN	Transgelin	Cancer development inflammation	1.58	−52.4

CON, control group; LP, low-protein group.

**Table 4:** Expression profile of DE muscle-related genes.

**Gene symbol**	**Gene name**	**category**	**logFC (LP vs. CON)**	**Ln *(p*-value)**
DES	Desmin	Muscle contraction	−1.57	−252.1
LMOD1	Leiomodin-1	Muscle contraction Muscle cell proliferation	1.31	−33.8
SYNM	Synemin isoform X1	Muscle contraction	2.47	−46.5
CNN1	Calponin-1	Muscle contraction Actin binding	1.97	−37.2
TPM2	Tropomyosin beta chain	Muscle contraction Actin binding	1.17	−132.2
MYH11	Myosin-11 isoform X2	Muscle contraction Actin binding	2.16	−97.6
MYL9	Myosin regulatory light polypeptide 9 isoform X1	Muscle contraction Actin binding	2.11	−91.6
MYLK	myosin light chain kinase, smooth muscle	Muscle contraction	2.26	−106.27
TPM1	Tropomyosin alpha-1 chain isoform X9	Muscle contraction Actin binding	1.15	−81.1
PDLIM3	PDZ and LIM domain protein 3 isoform X1	Muscle cell proliferation	1.21	−28.9
RBPMS2	RNA-binding protein with multiple splicing 2	Muscle cell proliferation	2.31	−8.97
DMPK	Myotonin-protein kinase isoform X1	Muscle cell survival	1.52	−39.0
LDB3	LIM domain-binding protein 3 isoform X1	Muscle development	1.87	−34.3

CON, control group; LP, low-protein group.

### 3.4. Microbial diversity and composition

**Figure 1:**
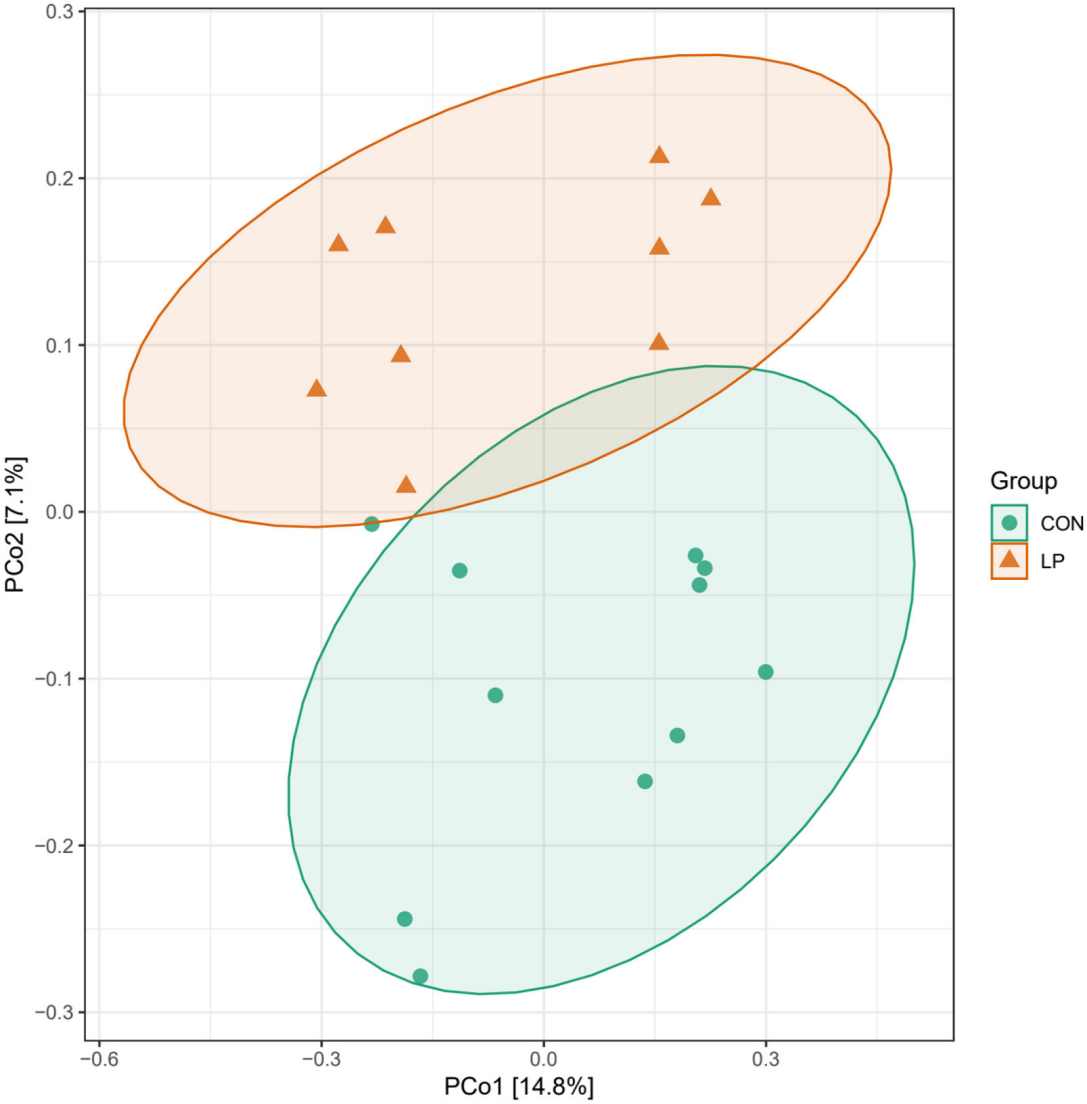
Principal coordinate analysis of ruminal mucosal microbiota of goats fed with LP vs. CON diets. LP, low-protein group; CON, control group.

**Figure 2:**
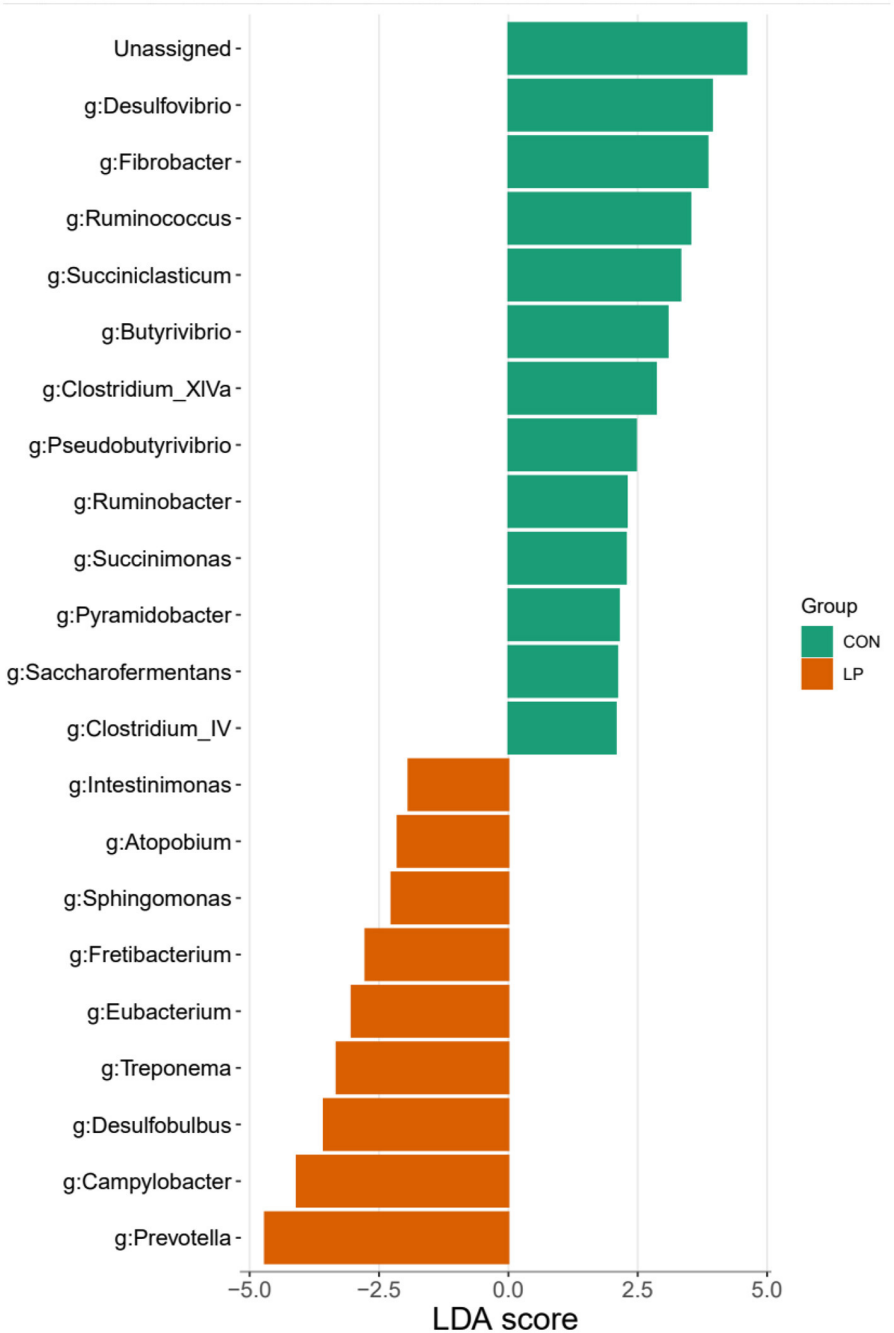
Differential microbe of ruminal mucosa in goats fed with CON vs. LP at the genus level. LP, low-protein group; CON, control group.

The alpha diversity analysis in ruminal mucosa showed lower ACE (*P* = 0.013), Chao1 (*P* = 0.015), and Shannon (*P* = 0.031) diversity indexes in the LP group than in the CON group, while the Simpson index showed no significant differences (*P* = 0.098, Table 5). Beta diversity analysis showed a clear distance separation between the LP and CON groups, and the major variance observed between these two groups was 14.8% (Figure 1). To explore these findings, we performed a LEfSe analysis to identify the differential microbial taxa between the LP and CON groups. The results showed that 9 microbial genera, namely, *Intestinimonas, Atopobium, Sphingomonas, Fretibacterium, Eubacterium, Treponema, Prevotella, Desulfobulbus*, and *Campylobacter* were enriched in the LP group while 11 microbial genera, namely, *Desulfovibrio, Fibrobacter, Succiniclastium, Saccharofermentans, Butyrivibrio, Ruminococcus, Ruminobacter, Succinimonas, Clostridium-XIVa, Pseudobutyrivibrio*, and *Pyramidobacter* were enriched in the CON group (Figure 2, LDA score >2).

**Table 5:** Alpha diversity analysis of ruminal mucosal microbiota in goats fed with LP vs. CON diets.

**Items**	**Treatments**		**SEM**	***p-*value**
	**CON**	**LP**		
Chao1	6,460.43	5,584.84	791.57	0.013
ACE	6,522.94	5,662.03	820.71	0.015
Shannon	6.81	6.35	0.52	0.031
Simpson	1.00	0.99	0.003	0.098
Coverage	0.96	0.97	0.005	0.006

LP, low-protein group; CON, control group.

### 3.5. Interactions between bacterial biomarkers and DEGs

**Figure 3:**
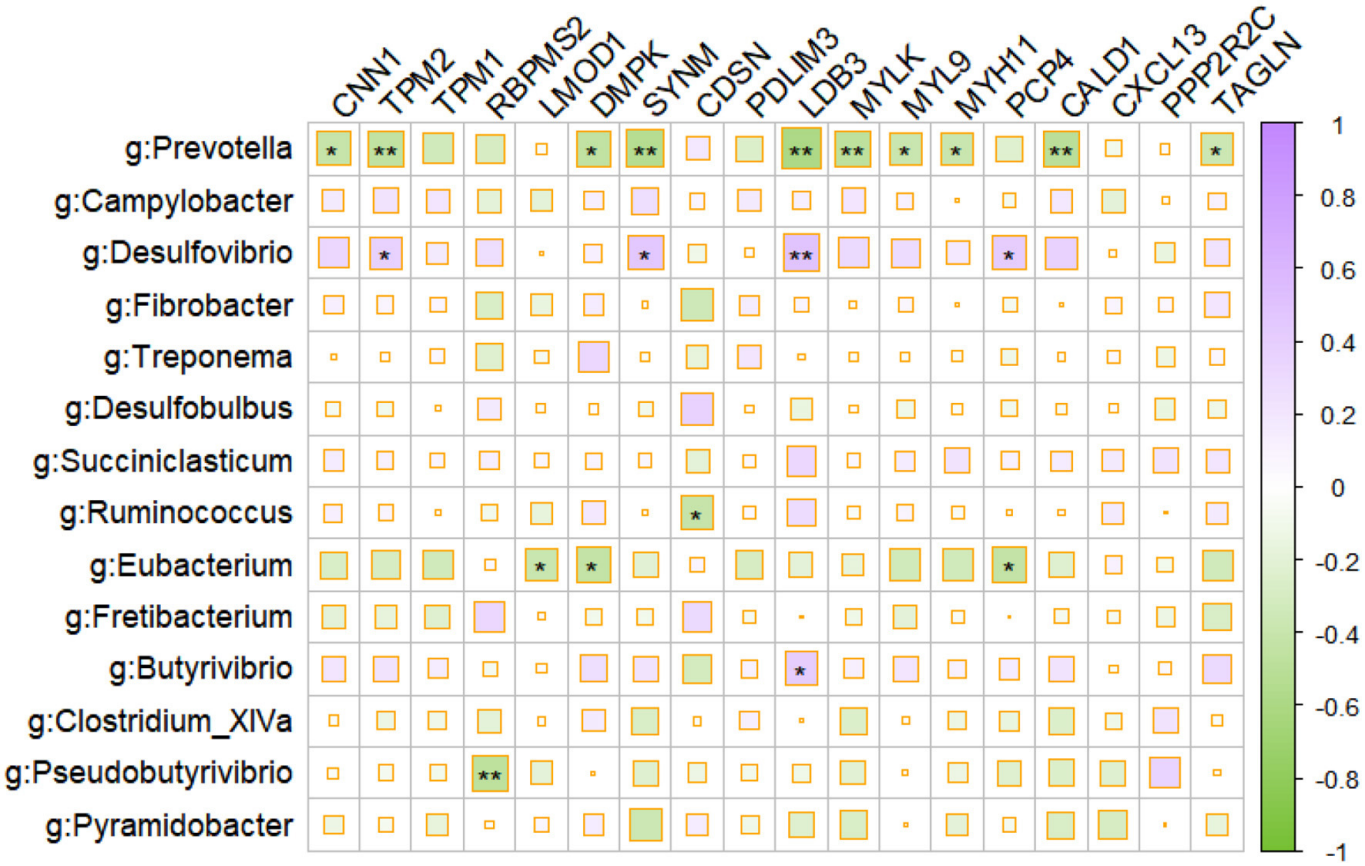
Correlation plot depicting gene–microbe correlations. Color and size of the squares indicate the magnitude of the correlation, asterisks indicate the significance of the correlation (**indicates a *p*-value of < 0.05, and *indicates a *p*-value of < 0.1).

We investigated the correlations between differentially expressed genes (DEGs) and the prominent microbes identified by the LEfSe analysis, in order to gain insight into the interaction between the host and microbiota in the rumen (Figure 3). Using Spearman correlations, we identified a strong negative association between the majority of immune and muscle-related DEGs and the prominent microbes, such as *Prevotella* and *Eubacterium*.

## 4. Discussion

It is usually considered that the protein ingested from the diet is converted into NH_3_-N in the rumen and then utilized by the ruminal microbiota to synthesize microbial protein (MCP). In addition to MCP, peptides and free amino acids digested from the dietary protein and synthesized by the ruminal microbiota (Russell et al., 2009) could be absorbed in the rumen mucosa (Poole et al., 2003). Not surprisingly, low-protein content in the diet induced a decline in the ruminal mucosal free amino acid concentrations, especially branched-chain amino acids (BCAAs, such as valine and isoleucine) and non-essential amino acids (NEAAs, such as glutamic acid and aspartic acid). It has been reported that the scanty of glutamate and BCAAs could induce the suppression of cell proliferation and protein synthesis (Sancak et al., 2010; Li et al., 2016). Hence, it is reasonable to observe a decline in the morphology of the rumen papilla.

Another notable change in response to the LP diet was genes involved in the muscle, which was likely the result of cells from the muscle layer that remained adhered to the dissected mucosal samples. Previous studies indicated that feeding a low-protein diet to mammals induces a decline in muscle layer thickness and muscle destruction in the GIT of animals (Peng et al., 2017). Although *TMP1, TMP2, DMPK, PDLIM3, RBPMS2*, and *LDB3*, which were reported to engage in muscle repairment (Jauvin et al., 2017), muscle cell proliferation (Notarnicola et al., 2012; Yin et al., 2020), and muscle regeneration (Knight et al., 2003), were upregulated in LP diet, the upregulation of *MYLK, MYH11*, and *MYL9* may induce muscle relaxation and GIT movement retention (Iwasaki et al., 2001) and finally impose negative effects on nutrient digestion and immune homeostasis (Kashyap et al., 2013). Muscle repairment and muscle regeneration are closely related processes. Muscle repairment refers to the physiological mechanisms involved in restoring damaged muscle tissue, while muscle regeneration refers to the process by which new muscle cells are formed to replace damaged or lost muscle tissue (Notarnicola et al., 2012; Yin et al., 2020). Movement retention is linked to both muscle repairment and muscle regeneration. When muscles are properly repaired and regenerated, movement retention is improved, allowing individuals to maintain their ability to perform various physical activities (Iwasaki et al., 2001). Additionally, the decline in muscle layer thickness and muscle destruction in the rumen may account for the downregulation of *DES*, which was considered the major component of muscle fibers in the LP group (Cizkova et al., 2009). The *LMOD1*, which promoted muscle relaxation (Nanda et al., 2017), was also upregulated in the ruminal mucosa and involved in the procedures of rumen contraction shunt.

In addition to the function of muscle contraction, the *MYH11* also plays vital roles in a series of biological processes, such as intracellular signaling transduction and cell adhesion (Derycke et al., 2011; Bowers et al., 2012). It has been reported that the promotion of *MYH11* expression was related to cell mobility and would cause the disruption of organ integrity and barrier function (Poninska et al., 2022). As anticipated, we observed a decline in the width and surface area of the ruminal papilla. Synchronously, LP inclusion resulted in the upregulation of *PCP4*, which played a vital role in the procedure of pathological microbiota infection resistance (Ge, 2003; Sui et al., 2021). It is not surprising to find that *CALD1* and *TAGLN*, which promoted inflammation, were upregulated in the LP group, implying an elevated inflammation risk in the GIT (Shen et al., 2010; Liu et al., 2021). Notably, the procedures of antigen presentation to immune cells were upregulated *via* the upregulation of *CXCL13* (Ohmatsu et al., 2007; Wang X. B. et al., 2007), to enhance the immune system and finally alleviate the negative effect of limited protein supply for goats.

Feeding LP diet to goats decreased the ruminal microbiota diversity, which was in line with previous reports in beef steers (Wang et al., 2017). It is noteworthy that several pathogenic bacteria genera propagated in ruminal mucosa during LP inclusion, namely, *Fretibacterium, Treponema, Intestinimonas, Sphingomonas*, and *Campylobacter* (Brooks et al., 2017; Gubert et al., 2020; Fan and Pedersen, 2021). These pathogens possessed the capacity to produce toxic substances such as endotoxin in the GIT (Zhao et al., 2013; Fan and Pedersen, 2021), thereby aggravating the above-mentioned disruption of immune homeostasis in the ruminal mucosa. In addition, the higher proportion of starch as a primary carbohydrate source in the LP group may provide a more suitable growth and metabolic environment for *Fretibacterium* and *Campylobacter*. This could potentially lead to a relatively higher abundance of *Fretibacterium* in the LP group.

Additionally, the results of our analysis revealed a significant negative association between DEGs related to immune response and muscle function and the prominent microbes, such as *Prevotella* and *Eubacterium*, in the rumen, suggesting that these microbial taxa may have a suppressive effect on the immune system and muscle development of the host. Meanwhile, the surge of H_2_S-producing *Desulfobulbus* in the mucosal microbiota during LP intervention (Cabrera et al., 2006) might also contribute to the disruption of immune stability in the ruminal mucosa. Intriguingly, LP inclusion declined the abundances of generally accepted carbohydrate-degrading bacteria in the ruminal mucosa, namely, fiber-degrading *Fibrobacter* and *Ruminococcus* (Yeoman et al., 2021), acetate producers *Desulfovibrio* and *Saccharofermentans* (Rettenmaier et al., 2021), succinate producers *Succinimonas* and *Pyramidobacter* (Gilbert et al., 2018), propionate producers *Succiniclastium*, and butyrate producers *Butyrivibrio* and *Pseudobutyrivibrio* (Pidcock et al., 2021). These highlighted the significance of ruminal microbiota in short-chain fatty acid (SCFA) production for energy supply and indicated a decline of carbohydrate fermentation in ruminal mucosa during LP intervention. Since the SCFAs not only act as the energy source for ruminants but also act as vital signaling molecules in maintaining immune homeostasis (Yao et al., 2022), the decline in the abundance of VFA-producing microbiota in the LP diet may also contribute to the host mucosal immune disruption.

## 5. Conclusion

Feeding LP diets to goats significantly decreased NEAA and BCAA concentration in the rumen mucosa, restrained the ruminal papilla growth, altered the gene expression related to muscle contraction, and depressed the gene expression related to immune homeostasis. Moreover, the low-protein diet also induced a decline in the relative abundance of carbohydrate-degrading microbiota while the promotion induced the abundance of pathogenic microbiota in the ruminal mucosa. Taken together, feeding LP diets to goats imposed severe challenges to goats as a result of the combination of pathogenic microbiota enrichment and the disruption of ruminal immune homeostasis.

## Data availability statement

The datasets presented in this study can be found in online repositories. The names of the repository/repositories and accession number(s) can be found below: https://ngdc.cncb.ac.cn/gsa—CRA011528 and CRA011517.

## Ethics statement

The animal studies were approved by the Animal Care Committee, Institute of Subtropical Agriculture, Chinese Academy of Sciences. The studies were conducted in accordance with the local legislation and institutional requirements. Written informed consent was obtained from the owners for the participation of their animals in this study.

## Author contributions

JW and ZT designed the research. CT and JJ performed the research and analyzed the samples. CZ and CT contributed intellectually to the analysis and interpretation of the data. CT, JW, and ZT wrote the manuscript. All authors read and approved the final version of the manuscript.
