# Supplementary material for: The epithelial transcriptome and mucosal microbiota are altered for goats fed with a low-protein diet
Source: Front Microbiol. 2023 Sep 4;14:1237955. doi: 10.3389/fmicb.2023.1237955 (PMC10507412; doi:10.3389/fmicb.2023.1237955)
Supplement: Supplementary file 1 [file Table_1.DOCX]

**Table S1**

Ingredients and chemical compositions of the control and low-protein diets (DM basis)^a^

| Items | Treatment | |
| --- | --- | --- |
|  | Control | Low-protein |
| Ingredients, g/kg |  |  |
| Rice straw | 700 | 700 |
| Soybean meal | 150 | 0 |
| Corn meal | 82 | 230 |
| Wheat bran | 29 | 29 |
| CaCO_3_ | 1.0 | 1.0 |
| CaH_2_PO_4_ | 3.0 | 5.0 |
| Soybean oil^b^ | 10 | 10 |
| NaCl | 5.0 | 5.0 |
| Premix^3^ | 20 | 20 |
| Chemical composition, g/kg | | |
| DM, g/kg as fed | 962 | 959 |
| OM | 873 | 877 |
| CP | 108 | 55.2 |
| NDF | 498 | 509 |
| ADF | 284 | 289 |
| Starch | 115 | 207 |
| GE, MJ/kg | 16.8 | 16.9 |

^a^ ADF, acid detergent fiber; CP, crude protein; DM, dry matter; GE, gross energy; NDF, neutral detergent fiber; OM, organic matter.

^b^ Soybean oil was composed of 16% saturated fatty acids, 25% monounsaturated fatty acids, and 59% polyunsaturated fatty acids.

^c^ Premix was formulated to provide (per kg of premix): 400 g NaHCO_3_, 2 g Fe, 1 g Cu, 0.01 g Co, 0.05 g I, 6.6 g Mn, 4.4 g Zn, 0.003 g Se, 333 mg retinol, 5 mg cholecalciferol, 838 mg α-tocopherol.
